# Individualization of FSH Doses in Assisted Reproduction: Facts and Fiction

**DOI:** 10.3389/fendo.2019.00181

**Published:** 2019-04-26

**Authors:** Frank J. Broekmans

**Affiliations:** University Medical Center Utrecht, Utrecht, Netherlands

**Keywords:** FSH, ovarian response, live birth, safety, OHSS, ovarian reserve testing, dosage individualization, ovarian stimulation

## Abstract

The art of ovarian stimulation for IVF/ICSI treatment using exogenous FSH should be balanced against the relative contribution of other steps of the ART process such as the IVF-lab-phase and the Embryo-Transfer. The aim of ovarian stimulation is to obtain a certain number of oocytes, that will enable the best probability of achieving a live birth. It has been suggested that more oocytes will create a better prospect for pregnancy, but studies on the question whether the retrieval of a few oocytes less or more will make the difference are not clearly supportive for this mantra. Personalization strategies have been the subject of many studies over the past 20 years. Creating the optimal response in a patient in terms of live birth prognosis as well as OHSS risks may be based on information from the Ovarian Reserve testing using the Antral Follicle Count or Anti-Mullerian Hormone, the patient's bodyweight, the ovarian response in a previous cycle, and the dosage level of FSH. Taken together, steering the ovarian response into a supposed optimal range may appear difficult as the interrelation for each of these factors with the egg number is weak. Using OR testing for choosing FSH dosage, compared to a standard normal dosage of 150 IU, has been studied in several trials. Dosage individualization, in general, does not appear to improve the prospects for live birth, but the reduction in OHSS risk may be substantial. This implies that the use of high dosages of FSH in predicted LOW responders lacks any cost-benefit for the patient and may be abandoned, while in predicted HIGH responders, reduction of the usual dosage level of 150 IU may create better safety, provided that in case of an unexpected LOW response cancelation of the cycle is refrained from. In view of recent developments in using GnRH agonist triggering of final oocyte maturation, the trend could be that with the Antagonist co-medication system and a standard dosage of 150 IU of FSH, prior ovarian reserve testing may become futile, as safety can be managed well in actual HIGH responders by replacing the high dose hCG trigger.

## Introduction

### The “ART” of Assisted Reproduction

Infertility is a disease state with potential profound consequences for the quality of life of both women and men. Reproduction is one of the key elements of life and failing to create offspring may lead to lifelong mental and physical health problems. Also, couples faced with infertility are frequently subjected to long-lasting, time consuming, and agonizing treatment schedules, living often between hope, and fear, and frustration. The development of IVF as a tool for solving problems such as tubal disease, severe male factor, anovulation states, and even, although not convincingly proven, conditions like ill-explained infertility, has brought enormous potential to the infertility treatment armamentarium.

Very soon after the development of the IVF technology, the single oocyte system was replaced by the art of ovarian stimulation in order to obtain multiple oocytes. This was aimed at solving two problems: one was the elimination of the risk of having no oocyte at all. The other was the urge to improve efficiency, by obtaining several embryo's and by replacing more than one in order to yield the highest possible probability of a live birth. Ovarian stimulation has thereby become one of the cornerstones of the IVF treatment, next to the *in vitro* handling of gametes and embryos, and the embryo replacement process.

The relative contribution to the overall success of IVF from the ovarian stimulation phase is difficult to assess. Many years of research have aimed at optimizing this specific phase. Issues have been addressed ranging from using urinary FSH products or recombinants, using high or low FSH dosages, triggering with urinary or recombinant, high or low dosage of hCG, adding LH or LH like activity to the FSH as principal drug, management of high, and low responders, adding medication to improve antral follicle availability, etcetera. At the same time, debates have been kept on beliefs like “the more (oocytes) the better,” less (mild stimulation) is more (quality), “normal (8–15 oocytes) is the best,” and “we need eggs, not ALL the eggs.” It seems that agreement on how ovarian stimulation could contribute to the best probability of success is far from settled.

### Folliculogenesis

**Figure 1:**
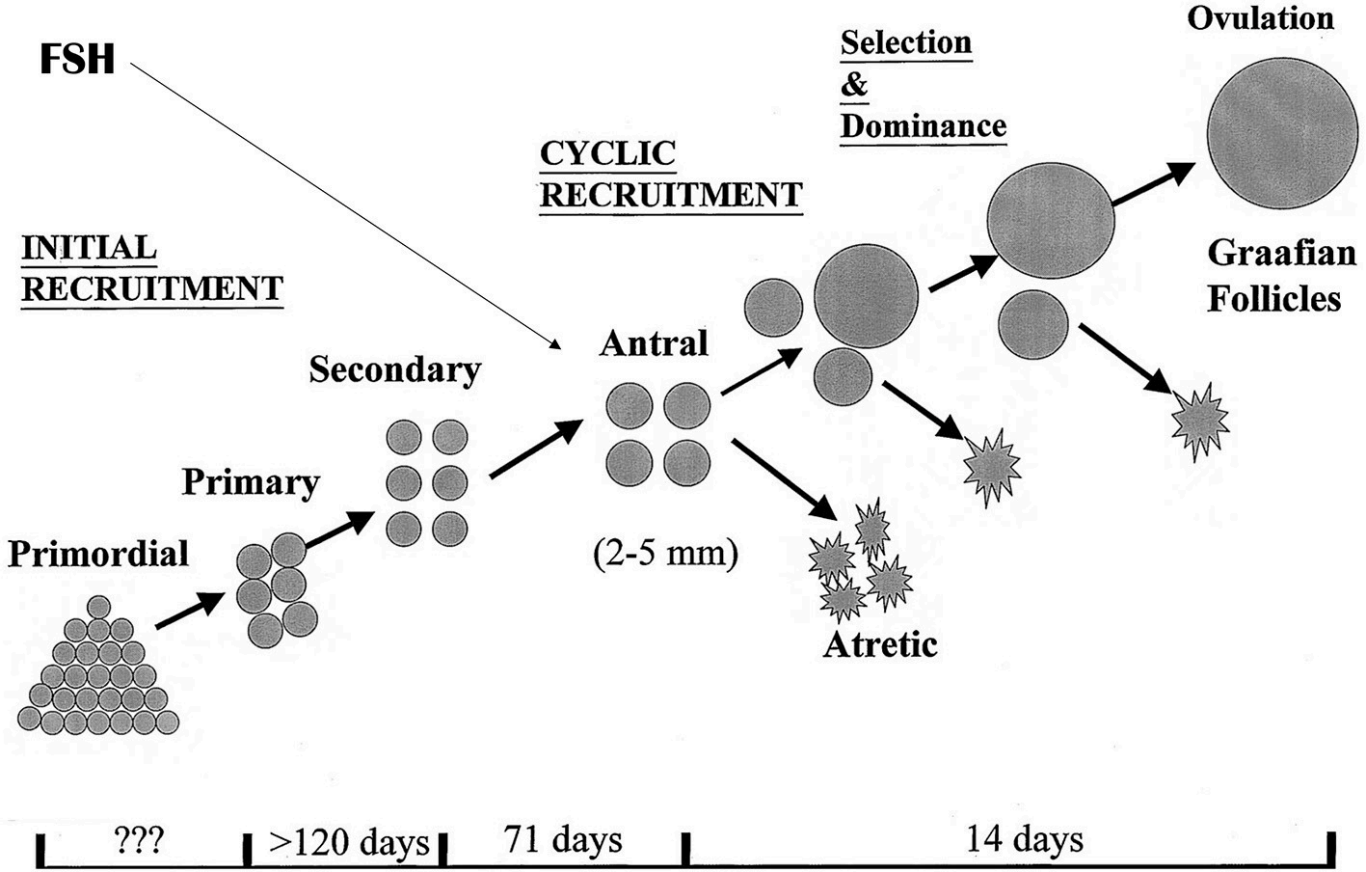
Folliculogenesis in the human ovaries. The antral stages of development provide a continuous target for exogenous or endogenous FSH to drive all or part of the present follicles into dominant follicle growth. It is demonstrated that the ovaries have initial, continuous recruitment with continuously filling, and emptying the pool of antral follicles, a process that is highly independent of control by pituitary hormones. Only during reproductive years, cyclic recruitment from the antral follicle pool occurs resulting in the ovulatory menstrual cycle.

Complex as it seems, the endocrine background for ovarian stimulation is quite straightforward. FSH levels must become elevated above the level that in the normal menstrual cycle will help to select and grow ONE single follicle, out of a group of antral follicles presenting in the FSH “window.” During this window period, levels of FSH surpass a certain threshold above which follicle granulosa cells become responsive and start to enhance proliferation, leading to expansion of the granulosa cell mass and the follicle fluid volume This will typically lead to the development of only one follicle, while other potential responsive antral follicles are destined for atresia, as a result of selection mechanisms that are still not fully understood (Figure 1). In surpassing the FSH threshold to a greater extent and for a longer period of time, more than one of the antral follicles will become capable of entering the dominant follicle development stage, with the ultimate opportunity of triggering the ovulation process and harvest the eggs within these follicles. Apart from administering FSH as an exogenous drug for the maturation of more than one follicle, other compounds such as selective estradiol receptor blockers, or steroid biosynthesis inhibitors may yield the same effect: increase and prolonged FSH exposure, albeit from an endogenous source.

### Pharmacokinetics: FSH Levels

For the drug FSH it has become clear that the one-compartment model with first-order absorption and a transit model for adding a delay in the absorption best describes the process of drug distribution and elimination in the body. This model principally assumes that the human body acts like a single, uniform compartment. When FSH is given in the form of a subcutaneous bolus, the entire dose of the drug enters the bloodstream after a short lag phase and distributes via the circulatory system to potentially all the tissues in the body. The modeled distribution implies that bodyweight, but not other potential confounders such as subject's age, affects the volume of distribution and clearance rate of the FSH medication. Both these effects, however, are small, with substantial variation in FSH serum levels after a standard dosage within bodyweight classes (1).

### Pharmacodynamics: FSH Dosage and Number of Oocytes

**Figure 2:**
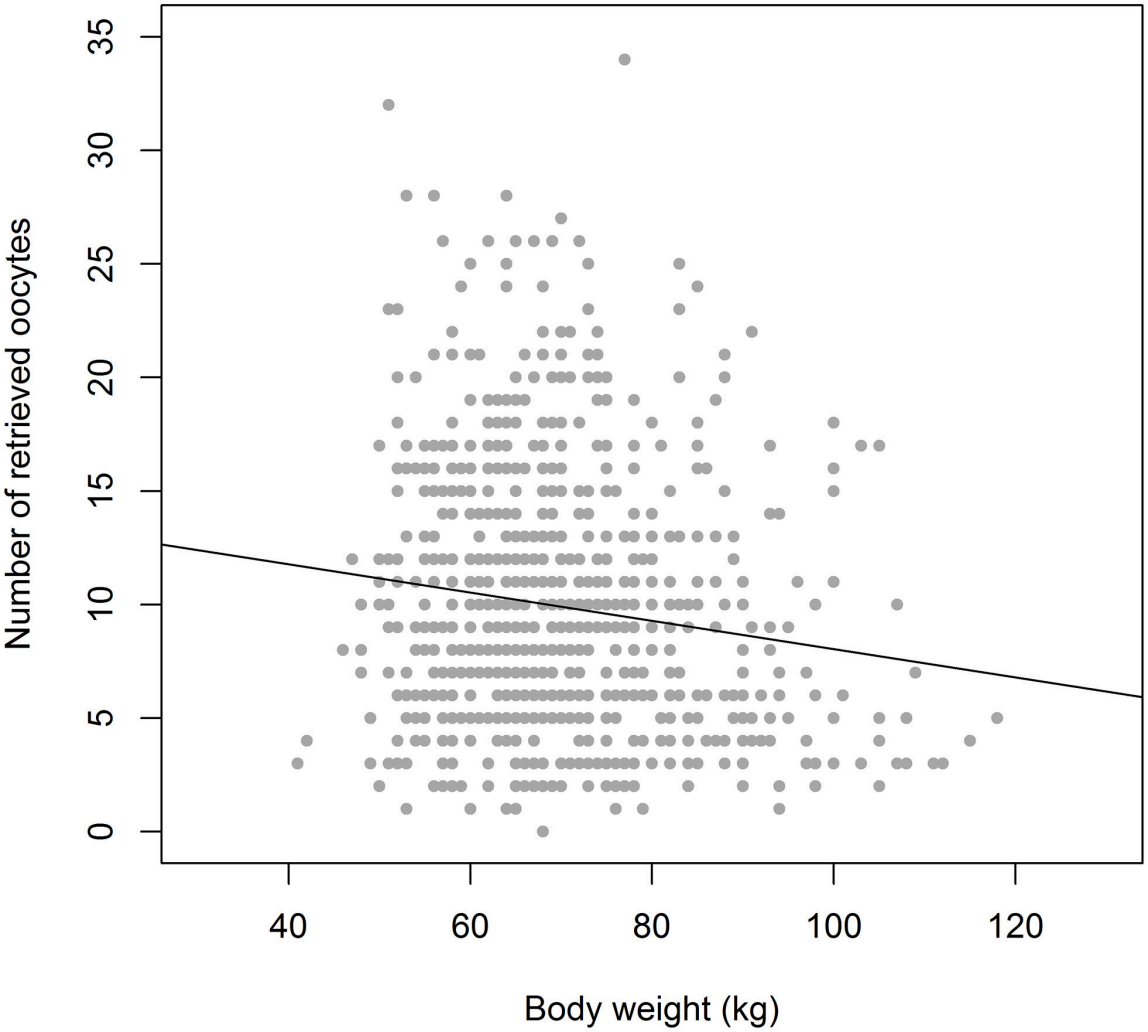
The relation between Bodyweight and Oocyte number in equal dosage (150 IU rec FSH) cases (*n* = 900), showing a weak correlation. With a weight of 60 kg the oocyte number ranges from 1 to 26. In the weight group of 90 kg the variation in oocyte number is not much different: 2–24. Drawn from the Optimist study database (18). It indicates that bodyweight may only have a weak role as a tool for dose assessment in ovarian hyperstimulation.

As indicated, the purpose of ovarian stimulation is to obtain at least one mature oocyte, and in most cases of prolonged supraphysiologic exposure to FSH, the response of the ovaries will be much more intense with a high degree of variation, ranging from 1 to 25 oocytes. The background for this variation may be multifactorial. The number of antral follicles present in the ovaries at any time will be the principal factor. However, under the assumption that these follicles may have different levels of sensitivity to FSH and may be at varying time points in their development through the antral stages, the level of exposure to FSH may be a second factor of importance. From a limited number of sources, it has become apparent that the exogenous FSH dosage will have some degree of positive relation to the oocyte yield, although it may only be true across a narrow range (from ~50 to ~225 IU per day). This relation is, however, far from precise, as actual serum levels of FSH, using a fixed daily dosage, may vary substantially across individuals, with a small contribution of body weight to this variation (2–4) (Figure 2). This all means that accurate steering of the oocyte number by the exogenous FSH dosing may not be a very reliable tool for obtaining a certain optimal oocyte number.

## Ovarian Response

### Response Categories

The level of oocyte yield has obtained a differential clinical appreciation, regards items such as success and safety. The “low” ovarian response defined as the yield of <4 oocytes is related to an unfavorable prognosis for live birth, although much of this poor prognosis is in fact dictated by female age and not by the low egg number *per se* (5). At the other side of the spectrum a high response, arbitrarily defined as obtaining more than 15 oocytes at pick up, will jeopardize safety for the patient and may even slightly limit the rates of live birth (6). It is therefore that many clinicians across the world try to foresee the ovarian response category in order to adjust the stimulation protocol with the expectation that the ovarian response can be brought into a “normal range” (5–15 oocytes).

### Factors That Predict Ovarian Response

Prediction of ovarian response category today is mainly applied by using the Antral Follicle Count by transvaginal ultrasound examination or the serum AntiMullerian Hormone level in the early follicle phase. Both relate to the number of antral follicles present at any time in the ovaries. These are the source for the number of dominant follicles that could grow in result to the application of exogenous FSH. As such, these two ovarian response tests (ORTs) have become the standard test for ovarian response prediction, although factors such as female age and, possibly, bodyweight may add to this predictive information.

Both tests may be affected by factors that may make response prediction less reliable. For the AFC ultrasound equipment quality and interobserver variation may be troublesome, as is the exact category of follicles: are only sizes of 2–6 mm or all follicles sized 2–10 mm counted on one of the first days of the cycle (7, 8). AMH assays have been under intense development over the past 15 years, leading to quite some inter-assay variation in results. With the advent of well-controlled automated assay systems many of the procedure problems have now been dealt with, although current available systems may not perfectly overlap (9, 10). It may therefore be noted, that AFC or AMH based predictions will be false positive in some 15–20% of cases, while only 60–70% of truly “out of the normal range” responders will be identified. Basing the FSH stimulation dosage on such predictions therefore may be imprecise practice from the start. Whether this reliability problem arises from imprecise response categorization by the ORT or relates to variation in the ovarian response within the patient to an equivalent dosage of FSH is not fully clear.

Parallel to this, FSH receptor polymorphisms have been long considered as welcome new attributes for response prediction (11). The Asn/Ser allelic variant may reflect a higher FSH sensitivity of follicles, leading to a better and more rapid ovarian response compared to the other two SNP variants. This differential FSH sensitivity may well be overcome by slightly lower or higher FSH dosages, but seems not to impact on live birth rates (12–14). Meaningful application of FSH receptor genomics in dosage personalization is still awaited.

### Factors That Predict Success

Success in Assisted Reproduction is defined as the occurrence of an ongoing pregnancy, leading to a healthy live-born, as a result of the IVF procedure. As indicated, the relative contribution of the *ovarian stimulation phase and oocyte retrieval* to this major outcome is not really known. In principle, the *laboratory phase*, with characteristics such as fertilization rate, embryo development rate, and embryo implantation rate, is an important part of the ART process, and must be under rigorous quality control. Also, the *luteal phase with the embryo transfer*, with the endocrine management of endometrium development and timing, and the deposition of the embryo in the uterus with only indirect and incomplete information on the correct “arrival” of the embryo, will contribute greatly to the outcome of the three-step process.

It is assumed that the quality of the oocytes that arrive in the IVF laboratory after follicle aspiration is the important factor. Good quality oocytes handled under optimal laboratory conditions and subsequent good quality embryos placed smoothly and well-timed in the uterine cavity, create the highest chances for having a baby from the ART cycle.

The question is then: will the approach in the stimulation and egg retrieval phase make a difference for the oocyte quality? Many clinicians today follow the idea that more oocytes will lead to a better outcome, especially regarding live birth rates. Such belief is probably not supported by evidence, but strongly suggested by retrospective studies (6, 15). In these studies, the individual patient profiles may be much more relevant than the number of obtained oocytes. The real question here is whether retrieving seven oocytes where potentially the patient could have had 11, creates a disadvantage, or reversely, whether a patient would have a benefit from creating 12 instead of the eight oocytes she obtained in a previous stimulation cycle. The answers here should come from randomizing these two hypothetical patients, and as this is impossible, to rely on group-randomized studies. Such studies seem to indicate that getting oocytes may be more important than striving for a maximal response (2, 7–9). Still, we struggle with a lack of knowledge on whether the hierarchy among the cohort of antral follicles that is capable of responding to elevated FSH levels from exogenous source, is such that the most sensitive follicles will provide the best oocytes in this cohort. From studies where only part of the recruitable follicles are driven into dominant growth and subsequent oocyte retrieval, it has been suggested that this will not create a lower number of good quality eggs compared to maximal stimulation where all follicles present in the FSH sensitive window are captured (2, 16, 17). More specifically, the relation between dosage of recFSH and ovarian response was studied in a randomized design. With increasing dosage, increasing numbers of oocytes were obtained, both in “low” as well as in “high” predicted responders. However, the cumulative rates of ongoing pregnancies per FSH dosage group, from fresh and frozen replacement cycles, revealed no better outcomes with increasing number of oocytes harvested (2).

Finally, knowing oocyte quality beforehand is to date not possible. We have a clear knowledge gap regarding the question which quality level is present for the oocytes present in the follicle cohort of a specific woman, as well as the quality of the monthly ovulated oocyte. The same is true for a woman entering an ART programme, where we would wish to know whether this woman is a good, poor, or moderate egg quality carrier. It is only after oocyte retrieval that some of this quality information becomes unveiled. More specifically, the retrieval of immature oocytes at aspiration, after well-timed ovarian stimulation and triggering of the ovulation process, does indicate a quality problem that is easily recognized in the lab. The vast majority of oocytes, however, will be mature and have succeeded in getting into the metaphase II stage. How to identify overall quality, and more interestingly, the individual competence of these oocytes to create a viable embryo after fertilization and to move on to the subsequent birth of a baby. Only small pieces of information have recently emerged on factors that may indicate quality in the *in vitro* stage and could become useful as a testing device, such as IL7 (19) and EGF (20). Whether such tools will help only in selecting the best oocyte or may also assist in optimizing the *in vivo* oocyte-follicle maturation during ovarian stimulation remains to become reality.

## The Role of FSH Dosage in Optimizing Outcome

### What Is the Normal FSH Dosage?

From the limited number of studies that have tried to study the dose response relationship for the drug FSH (2, 2, 21, 22) it has become clear that going from only a single dominant follicle to a maximal ovarian response the FSH dosage needs to be raised from ~50 to 225 IU daily. In order to obtain a reasonable number in between only one and the maximum, a daily dosage of 150 IU is often promoted and in fact adopted as an empirical “normal” dose. Such dosage will allow for obtaining an optimal or “normal” number of 8–15 oocytes in a large part of the ART patient population. However, with this dosage a subset of patients will produce either a Low or High response, and for reasons outlined above, clinicians are keen on trying to prevent such conditions, amongst other by FSH dosage individualization. In fact, the belief is such that with effective correction of the Low responder into a Normal responder the live birth rates will improve. Also, the production of a Normal response in High responders will create a better safety profile, without jeopardizing the outcome live birth. Although such High responder management is very likely to be a real improvement for the patient (23), the low responder may not have any benefit from FSH dose adjustments. This may be true for predicted low responders, as they have no additional follicles available, but also for unexpected low responders, who will have no better prospects in spite of a higher egg number (3, 24).

Today, FSH dosage individualization is based on two components. First, there is a need for an Ovarian Response Test that can predict a woman's response when given a particular dose of FSH. Second, there must be some dose-response relationship, enabling manipulation of the response through adaptation of the dose. With regard to prediction of response, studies have reported that ORT can be used to predict ovarian response to stimulation, with AMH and AFC being superior to bFSH (25–27). Thereby, the effects of FSH dose adjustments in ovarian response categories could be studied.

### Is There a Best FSH Preparation?

Compounds containing FSH as primary component are urine derived mixtures of FSH and LH, sometimes enriched with human chorion gonadotropins, urine derived FSH only preparations, recombinant technology based FSH preparations with or without added recombinant LH, and slow release, long acting modifications. Many efforts have been undertaken over the past three decades to demonstrate the benefit of one preparation over the other. Issues like FSH dosage stability and added LH (or hCG) activity for full sustained endocrine support of the follicle have formed most of the backgrounds to propel research, next to cost efficacy needs.

Looking into the current literature there is no evidence of a preference for any of the compounds available today (28–30). This may also be true for application in specific subgroups of patients such as low responders (28, 31), where neither LH/hCG enriched, nor long-acting FSH only compounds have made a difference (32). The only category with a specific and obvious need of ovarian stimulation with both FSH and LH are patients with a hypothalamic amenorrhea.

### Higher Dosages in Predicted Low Responders

Several randomized controlled trials have demonstrated that ORT based individualized dosing of FSH will not alter the fate of the predicted low responder. Specifically, in predicted poor responders the actual occurrence of a poor response will mean that the couple is in a prognostic unfavorable category, although female age may be an important additional value for the real prognosis (5). The prognosis for live birth in young predicted low responders may indeed be three times as good compared to old predicted poor responders (33). Thus, the combination of low AMH or AFC, the actual first cycle poor response, and female age may help to decide whether continuation of the ART treatment is really feasible. This theme has been clearly addressed by the POSEIDON group, where both the prior expectation regarding ovarian response, as well as the age of the patient will place her in distinct low responder groups, with potentially differing management and prognosis for live birth (34, 35). It is, however, clear that the use of extremely high dosages of FSH, such as 300–600 IU per day, will not make any difference for the patient, but do have undesirable effects on the costs of treatment (36).

### Lower Dosages in Predicted High Responders

The real gain of individualized FSH dosing could be the management of the hyper responding patient. Several studies have indicated that with the use of submaximal dosages of FSH a mitigated response of the ovaries can be obtained, without jeopardizing efficacy and with a clear improvement of the safety profile, in terms of measures needed to be taken to prevent the OHSS syndrome as well as the actual occurrence of the syndrome (2, 23, 37). This then could be considered as primary preventive management of the OHSS in predicted high responders. At the same time, we may consider whether a standard 150 IU dosage using an antagonist protocol, with the escape of GnRH agonist triggering and with a freeze all strategy as added option, may be the method of secondary OHSS prevention. Such a strategy would bypass imprecise dose picking based on ovarian response tests with moderate accuracy.

Regimens with GnRH antagonist LH peak prevention may, however, be impractical in view of planning issues regards the availability of the IVF laboratory in special cases such as ICSI-PGD or ICSI TESE. Here, OC pretreatment may affect the prognosis for live birth in antagonist cycles, while agonist co-medicated cycles do not seem to have these disadvantages. In the latter, only lower FSH stimulation doses and freeze all are available as safety tools. With lower dosages may arrive conditions where the hyper response is prevented, but in return a low response is observed (37). The fear by clinicians that the patient may then become disadvantaged by collecting fewer oocytes than believed to be optimal is not be supported by current evidence, and thus clinicians may reassure patients at this point (38–40).

The question then remains how ovarian response testing should be embedded in the ART programs. Should we screen every patient and only apply dose adjustments in predicted high responders? Or do we need to scout ovarian reserve status by applying the AFC as a screening test. This could select out patients to undergo an AMH assessment where in predicted high responders either reduced doses of FSH or standard dosing with antagonist co-medication protocols are applied.

For the subgroup of PCOS patients, FSH dosing studies are quite limited in number. In many cases previous cycles of low dose step up FSH ovulation induction will be of help in the right dose picking once entering an IVF programme, in order to manage the substantial risk of extreme hyper response in these patients. Dosages at which mono-follicular follicle growth is obtained may be increased by ~50 units to obtain multi-follicular growth (41). In order to remain within safety limits further parameters such as female age, BMI and AMH level may help in individualizing the dosage levels, which will typically be between 75 and 150 IU per day (42). Needless to state that a GnRH antagonist LH suppression regimen may be preferred in view of secondary options in OHSS prevention (43).

### ORT-Based Individualization Studies

**Figure 3:**
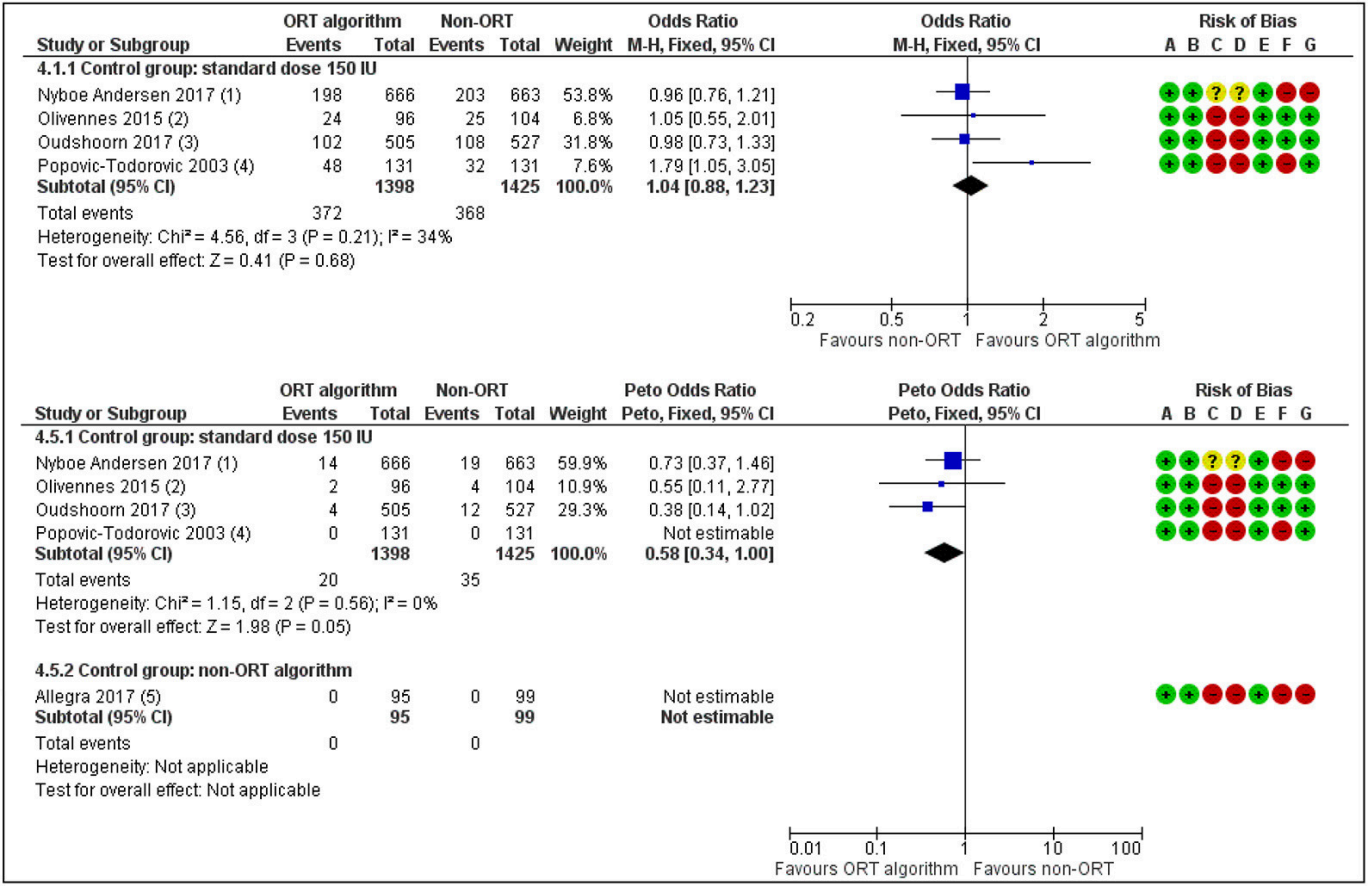
ORT-based vs. Standard dosing of FSH in IVF patients. Effects on Live birth or Ongoing pregnancy per woman randomized (upper panel) and on occurrence of Moderate or Severe OHSS. The individualized dosing has no beneficial effects on the outcome Pregnancy rates but does reduce treatment Risks [Redrawn form Lensen et al. (36)].

Five studies have so far studied the value of ORT based dosage individualization in the general ART population compared to a standard dose of 150 IU (23, 24, 36, 44–46). There is now moderate quality evidence for the absence of a difference between the groups in live birth rate (OR 1.04, 95% CI 0.88 to 1.23) (Figure 3). The incidence of moderate to severe OHSS was reduced when compared to a standard dose (OR 0.58, 95% CI 0.34 to 1.00) (Figure 3), but this evidence was of also of low quality. So, the promises that individualized dosing based on ovarian reserve markers would positively affect live birth rates in the ART program (46) have not been fulfilled. Yet, the possible gain of FSH dose individualization lies in the better grip on safety for the patient, albeit that dose management may not be the only way forward here. Studies that have compared the use of AMH with the AFC for dose individualization in the general ART patient (47), have not yielded any obvious difference in outcome live birth or OHSS rate. So, there is a strong need for trials recruiting specific patient groups for which a personalized approach would make the relevant difference compared to a standard approach. In such studies, both the added value at the level of oocytes number and good quality embryo's, as well as at the level of children's health need to be considered.

## Summarizing Conclusions

For many years we have held the belief that more oocytes will produce better outcome in terms of live birth rate. The current evidence from well-designed studies has helped us to separate fiction from facts. The facts are that we need more than one oocyte, preferably a number in the range of 8–15. Below that number, but specifically below five oocytes, prognosis for live birth will become jeopardized. Oocyte numbers over 15, and specifically over 20, are undesirable in view of the risk of OHSS occurring. Low responders cannot really be prevented by applying higher than normal dosages, while high responders may benefit from FSH dosage reduction, mainly for the safety issue. So, for that latter purpose, ovarian reserve testing and subsequent dose adjustments could be justified. The high responder patient, however, may also be served by the GnRH antagonist co-medicated stimulation approach: standard dosing with 150 IU, with the option of triggering final oocyte maturation by a GnRH agonist with or without deferred embryo transfer (48–50). With these facts together, we may find that the FSH dose individualization practice may become a realm of the past.

## Author Contributions

The author confirms being the sole contributor of this work and has approved it for publication.

### Conflict of Interest Statement

FB receives monetary compensation: Member of the external advisory board for Ferring BV, Netherlands. Member of the external advisory board for Merck Serono, Netherlands. Member of the external advisory for Gedeon Richter, Belgium. Educational activities for Ferring BV, Netherlands.
